# Knowledge and Compliance with Infection Prevention and Control Practices in Prosthodontic Procedures Among Dental Students and Professionals

**DOI:** 10.3390/healthcare12242536

**Published:** 2024-12-16

**Authors:** Lubna Alkadi, Fathima Fazrina Farook, Ibraheem Binmoghaiseeb, Yara Alyousef, Abdullah Alabdulwahab, Raghad Aljohani, Ali Asiri

**Affiliations:** 1 Department of Restorative and Prosthetic Dental Sciences, College of Dentistry, King Saud bin Abdulaziz University for Health Sciences, Riyadh 14611, Saudi Arabia; 2 King Abdullah International Medical Research Center, Riyadh 11481, Saudi Arabia; 3 King Abdulaziz Medical City, Ministry of National Guard Health Affairs, Riyadh 11426, Saudi Arabia; 4 Department of Preventive Dental Science, College of Dentistry, King Saud Bin Abdulaziz University for Health Sciences, Riyadh 14611, Saudi Arabia; 5 Restorative and Prosthetic Dental Sciences Department, College of Dentistry, Dar Al Uloom University, Riyadh 13314, Saudi Arabia; 6 Department of Epidemiology and Public Health, University College London, London WC1E 7HB, UK; 7 Department of Pediatric Dentistry and Orthodontics, College of Dentistry, King Saud University, Riyadh 11545, Saudi Arabia

**Keywords:** infection prevention, infection control, compliance, prosthodontics, knowledge assessment

## Abstract

**Background/Objectives**: Infection prevention and control (IPC) is essential to ensure the safety of dental personnel and patients. This study aimed to assess the knowledge and compliance of dental undergraduate students, interns, and postgraduate students with IPC measures in prosthodontic procedures. **Methods**: A cross-sectional observational study was conducted at the College of Dentistry, King Saud bin Abdulaziz University for Health Sciences, involving 216 participants selected using stratified random sampling. A validated questionnaire was used to assess knowledge and compliance. Statistical analyses, including the Mann–Whitney U test and Kruskal–Wallis test, were conducted to explore factors influencing knowledge and compliance levels. **Results**: Participants demonstrated a high level of IPC knowledge, with 93.55% correctly identifying the goal of infection control. However, gaps were noted, such as only 41.23% recognizing the recommended handwashing duration. Sex differences in knowledge were marginally statistically significant (*p* < 0.05), while academic level showed no significant association. Compliance was high in some areas, such as handwashing after treating patients (81.11%), but lower in others, such as disinfecting digital equipment between patients (36.87%). Higher self-confidence was significantly associated with greater knowledge scores (*p* < 0.05), while self-satisfaction with knowledge did not correlate with knowledge levels. **Conclusions**: This study highlights strong IPC measures knowledge and compliance during prosthodontic procedures among dental personnel, with some gaps in understanding and practice. Addressing these gaps through targeted training and standardized guidelines can further enhance safety and infection control in clinical settings, benefiting both patients and healthcare providers.

## 1. Introduction

Infection prevention and control (IPC) is a set of practical, evidence-based approaches designed to protect patients and healthcare workers from preventable infections [1]. The World Health Organization (WHO) provides tools, recourses and guidelines designed for national or facility level to achieve effective implementation of their IPC programs. Similarly, Centers for Disease Control and Prevention (CDC) and The Association for Professionals in Infection Control and Epidemiology (APIC) provide additional recommendations for IPC measures [2].

In Saudi Arabia, the General Directorate of Infection Prevention and Control (GDIPC) in the Ministry of Health (MOH) provides national guidelines on procedures and policies on IPC measures in healthcare facilities based on scientific references, such as the Gulf Cooperation Council Center for Infection Control (GCC-CIC), CDC, WHO, and APIC recommendations [3].

In dental clinics, IPC plays a vital role in ensuring the safety of operators, auxiliary dental workers, and patients by preventing the spread of infectious diseases within dental practices [4]. The knowledge and compliance of dental healthcare professionals with IPC measures are essential for maintaining a safe and hygienic environment.

The risk of disease transmission is an inherent part of dental practice, with various bacteria and viruses posing potential threats to dental students and workers. Data from multiple sources have identified herpes simplex virus, varicella-zoster virus, human immunodeficiency virus, hepatitis B, C, and D viruses, mycobacterium spp., and multi-resistant bacteria as potential transmission risks in dental settings [5]. Reports suggest that up to 70% of nosocomial infections are preventable by adhering to IPC guidelines and implementing safety precautions such as vaccination and following the post exposure protocols [4,6].

Cross-infection refers to the spread of infectious pathogens between staff members and patients in a healthcare setting [7]. Evidence indicates that disease transmission can occur through various pathways, including direct contact with bodily fluids (i.e., blood, saliva, or contaminated water), sharp and needlestick injuries, and contact with contaminated instruments and surfaces. The risk of cross-contamination is closely associated with the communication and contact between dental personnel and the dental laboratory. Numerous procedures and instruments involve direct contact with saliva and blood or the generation of aerosols and splatter, further increasing the risk [8]. Previous research has found that up to 88% of laboratory work received and sent to dental clinics was contaminated with mixed flora and microorganisms [9,10].

Studies have investigated the knowledge and attitudes of dental students toward IPC measures in prosthodontic procedures. The results of these studies have highlighted a lack of commitment in certain areas of the IPC practice. For example, Hakam et al. found that 73% of participants never disinfected their impressions [11]. Rehman et al. reported that 75.4% of participants did not disinfect rubber bowls between patients, and 56.9% did not disinfect facebows before sending them to the dental laboratory [12]. Alshiddi et al. assessed the occurrence of hazardous events such as sharp injuries and eye splashes, finding that these incidents occurred at rates of 57% and 30.2%, respectively [13]. On the other hand, a multi-center cross-sectional study conducted in 2020 in Jeddah, Saudi Arabia, evaluated the level of IPC practices in prosthodontic procedures. The authors reported that 80% of participants regularly disinfected their dental casts before sending them to the laboratory [14]. 

In prosthodontics, materials potentially contaminated with pathogenic bacteria are frequently transported between dental laboratories and clinics [15,16]. One common dental procedure that poses a risk of cross-contamination, particularly between patients and dental laboratory personnel, is the making and pouring of impressions [15,16]. Impressions, wax rims, and inter-occlusal records should always be disinfected before being sent to the dental laboratory [17]. The potential for cross-contamination with stone casts is particularly high in prosthodontics; therefore, these casts should be disinfected after each clinical and laboratory procedure [16]. Various disinfection methods have been described in the literature to reduce or eliminate the risk of cross-contamination, including immersion and chemical disinfection [18].

The current literature in Saudi Arabia indicates insufficient adherence to and awareness of IPC practices in prosthodontic procedures [13,14,15,16]. Studies consistently emphasize the need for targeted interventions, education, and training programs to enhance compliance with general IPC measures. The notable discrepancies in results observed across various studies highlight a significant gap between IPC protocols, participant knowledge, and their actual practice across different educational levels in dentistry, particularly in prosthodontic procedures. This gap suggests that while awareness of these may exist, consistent compliance remains a challenge. This is particularly important, considering the risks posed to patients, laboratory staff, and dental professionals [19]. Therefore, this study aimed to contribute to the existing body of literature by investigating the level of knowledge and compliance with IPC measures in prosthodontic procedures among dental undergraduate students, interns, and postgraduate students at the College of Dentistry (COD), King Saud bin Abdulaziz University for Health Sciences (KSAU-HS) in Riyadh, Saudi Arabia. The null hypothesis states that there are no differences in IPC measure knowledge and compliance among undergraduate students, interns, and postgraduate students.

## 2. Materials and Methods

### 2.1. Study Design and Setting

A cross-sectional observational study was conducted among fifth- and sixth-year undergraduate dental students, interns, and postgraduate students specializing in prosthodontics, restorative dentistry, advanced general dentistry, and family dentistry at the COD, KSAU-HS. Participants were included if they were enrolled in one of the specified academic programs who routinely perform prosthodontic procedures, consented to participate by completing the questionnaire, and provided complete responses. The exclusion criteria eliminated non-dental students, individuals outside the specified academic levels, participants who submitted incomplete or duplicate questionnaire responses, and those who declined to participate. Data were collected via a questionnaire distributed through Google Forms on various social platforms and through personal visits to clinics over a 5-month period, from January 2024 to May 2024. The questionnaire, adapted from previous studies, was designed to evaluate knowledge and compliance with recommended IPC guidelines in prosthodontic procedures [12,13,14,20,21,22,23]. Ethical approval for the study was obtained from King Abdullah International Medical Research Center (KAIMRC), Riyadh, Saudi Arabia.

### 2.2. Sampling Technique and Sample Size

A stratified random sampling technique was used. A sample size calculation was performed with a 95% confidence level, based on 73% overall knowledge, attitude, and compliance of IPC reported in a previous study, and a population size of 296 [24]. The required sample size for this study was determined to be 216 (consisting of 101 students, 78 interns, and 37 postgraduate students). 

### 2.3. Questionnaire Development

The content of the questionnaire was validated using the test–retest method. Approximately 10% of the study sample (consisting of 20 students from different academic levels) were invited to participate, with the questionnaire being re-administered after a two-week period. After this period, the results were compared using kappa statistics. Most items achieved a weighted kappa value of over 0.6, indicating substantial to great agreement. Six items achieved a weighted kappa value between 0.4 and 0.6, suggesting moderate agreement, and one item achieved a value of 0.37, indicating fair agreement. Necessary adjustments were made to items with moderate to fair agreement. Additionally, expert validation was conducted. 

### 2.4. Questionnaire Structure

The final questionnaire consisting of 47 items divided into six sections (Supplementary Materials): 

Section A: Demographic Data;

Section B: General Knowledge on Infection Control;

Section C: Knowledge of IPC Guidelines;

Section D: General IPC Compliance;

Section E: Prosthodontic IPC Compliance; 

Section F: Evaluation.

The first section includes two questions on the participants’ demographic information, including sex and academic level. The second section consists of ten questions about the participants’ understanding of infection control and its definition. The third section focuses on mandatory knowledge and adherence to IPC guidelines. Sections D and E consist of 30 items assessing compliance with IPC using a three-point Likert scale (1 = always, 2 = sometimes, 3 = never). The final section is dedicated to self-evaluation of overall knowledge, confidence, and satisfaction with IPC guidelines.

### 2.5. Data Collection

Participants were asked to complete the self-administered questionnaire. To address potential challenges in response rates, respondents were approached personally at their clinics and at convenient times, with multiple follow-ups to encourage participation.

### 2.6. Statistical Analysis

Data were analyzed using the NCSS 2020 Statistical Software Version 20.0.1 (NCSS, LLC. Kaysville, UT, USA). Descriptive analysis was conducted to understand the study sample. A scoring system was implemented, assigning 1 point for each correct response and 0 for incorrect responses. The total score for each participant was the sum of these individual scores. Proportions of the answers were tabulated for easy visualization, and a bar chart was created to illustrate correct and incorrect answers to the knowledge section. 

Due to the non-normal distribution of scores, the Mann–Whitney U test was conducted to examine the effect of sex on knowledge scores. In addition, the Kruskal–Wallis test was used to explore the relationship between knowledge scores and academic level. A Kruskal–Wallis H test was also conducted to assess differences in knowledge scores across four levels of self-reported confidence: excellent, very good, adequate, and limited. Furthermore, post-hoc analysis using Dunn’s test with Bonferroni correction was used to identify differences between these levels. A complete case analysis was done, where participants with missing data were excluded from the study. 

## 3. Results

The study included a total of 217 dental personnel, comprising 87 males (40.09%) and 130 females (59.91%). The participants were categorized into the following academic levels: D3 (fifth-year students) at 20.28%, D4 (final-year students) at 26.27%, interns at 36.41%, and postgraduate students at 17.05%. 

The participants generally demonstrated a high level of knowledge regarding IPC. For example, 93.55% correctly identified the goal of infection control, while 98.62% recognized that all patients could be potential sources of infection, regardless of their known medical status. However, knowledge gaps were identified in specific areas: only 53.92% correctly defined standard precautions, and just 41.23% were aware of the recommended duration for handwashing. These findings are summarized in Table A1 within Appendix A. 

Males had a mean knowledge score of 78.27 (SD = 13.25), while females had a mean score of 75.25 (SD = 13.77). The Mann–Whitney U test revealed a marginally statistically significant difference between males and females (U = 4780.5, Z = 1.9686, *p* = 0.049). In contrast, the Kruskal–Wallis test, which explored the relationship between knowledge scores and academic level, indicated no statistically significant difference across the different academic levels (H = 5.1652, df = 3, *p* = 0.160).

The level of compliance with various IPC practices varied among participants. High compliance was observed in some practices, such as handwashing after treating each patient (81.11%) and wearing gloves during patient treatment (90.78%). In contrast, lower compliance was noted in practices like replacing masks between different clinical procedures (31.34%) and providing patients with protective eyewear (40.55%). These results are detailed in Table A2 within Appendix A, which outlines the adherence of participants to specific IPC practices. In addition, the findings showed that 72 out of the 217 respondents (33.18%) reported experiencing a skin prick injury from a sharp instrument while treating a patient. Among those who experienced an injury, 58 (80.55%) reported the incident, while 14 respondents (19.44%) indicated they did not report the injury.

Further analysis focused on adherence to IPC specifically within prosthodontic clinics. The results showed high adherence in areas such as disinfecting rubber bowls between patients (87.10%) and sterilizing or disinfecting impression trays before use (77.42%). However, there were still areas requiring improvement, such as the disinfection of the scanning wand, mouse, keyboard, and touch screen between patients, where compliance was only 36.87%. The full list of items is outlined in Table A3 within Appendix A.

The questionnaire revealed varied levels of satisfaction among participants regarding their IPC practices. In total, 43.32% of respondents were completely satisfied with their practices, and 41.01% were mostly satisfied. A smaller group (13.82%) expressed being somewhat satisfied, while only a minimal number of respondents reported being slightly or not satisfied at all (0.92% each, with 2 individuals in each category). The Kruskal–Wallis H test revealed no statistically significant differences in knowledge scores among these satisfaction levels (H(4) = 7.909, *p* = 0.09496 uncorrected; H(4) = 8.2349, *p* = 0.0833 corrected). These results suggest that participants’ satisfaction with their practices does not necessarily correlate with higher knowledge scores.

When evaluating participants’ knowledge of IPC in prosthodontic procedures, respondents similarly demonstrated a high level of confidence. Out of the total respondents, 94 individuals (43.32%) rated their knowledge as excellent, and 91 respondents (41.94%) rated their knowledge as very good. Additionally, 30 participants (13.82%) considered their knowledge to be adequate. Only two respondents (0.92%) rated their knowledge as limited, and none indicated that their knowledge as insufficient. Overall, the data indicate a predominantly high level of satisfaction and confidence among respondents concerning their performance and knowledge in IPC within prosthodontic clinical settings. The Kruskal–Wallis H test revealed a statistically significant difference in knowledge scores across the confidence levels (H(2) = 6.5021, *p* = 0.0387 (uncorrected) and H(2) = 6.7698, *p* = 0.034 corrected)

Post-hoc analysis using Dunn’s test with Bonferroni correction identified significant differences between confidence levels. Specifically, individuals with excellent confidence had higher knowledge scores compared to those with limited confidence, with a Z-value of 1.9498; however, this difference did not reach statistical significance after Bonferroni correction (Z > 2.3940). 

Descriptive statistics indicated that individuals with excellent confidence had a mean score of 77.40 (SD = 12.89), those with adequate confidence had a mean score of 71.82 (SD = 16.60), and those with limited confidence had a mean score of 59.10 (SD = 6.43), with corresponding medians of 81.82, 72.73, and 59.10, respectively. These results suggest that higher confidence levels are associated with higher knowledge scores.

## 4. Discussion

Based on the results of our study, the evidence was insufficient to reject the null hypothesis; therefore, we accepted it. Our study showed comparable levels of knowledge between males and females, with mean scores of 78.27 and 75.25, respectively, and a statistically significant difference in the distribution of knowledge scores. This difference may be attributed to the higher proportion of female participants (59.91%). Additionally, knowledge score distribution was comparable across the different academic levels. Similarly, Shahadah and Halawani, in Madina and Jeddah, found that there were no associations between knowledge scores and academic level [14,25]. In contrast, international studies conducted in India and Egypt found that academic level was associated with IPC knowledge score [26,27]. This discrepancy may be explained by variations in IPC regulations, training structures, and educational frameworks between countries and institutions. For instance, certain nations may implement stricter IPC guidelines and provide comprehensive, mandatory training on these protocols as part of the curriculum, leading to greater knowledge differentiation across academic levels. On the other hand, countries or institutions with a more standardized approach to IPC education across all levels may not observe such associations. Furthermore, cultural factors, resource availability, and institutional priorities could also play a role in shaping the extent of IPC knowledge gained at different stages of academic progression. These contextual differences highlight the importance of tailoring IPC training to local needs while aligning with global best practices.

Most dental students are well-aware of the goals of IPC, with nearly all participants recognizing the importance of changing personal protective equipment (PPE) between patients, as shown in Table A1. However, notable knowledge gaps persist in core IPC practices, particularly in hand hygiene and the proper use of PPE during different clinical procedures, such as transitioning between extraoral and intraoral examinations on the same patient. Addressing these deficiencies requires targeted interventions, including the integration of comprehensive IPC training into the curriculum, conducting specialized workshops, disseminating educational materials such as posters and IPC policy guidelines throughout clinics, and ensuring the presence of dedicated staff to monitor compliance through regular rounds and provide constructive performance feedback.

Hand hygiene is considered the single most important, simplest, and least expensive means of reducing the prevalence of healthcare-associated infections [28,29,30,31]. According to a systematic review that included 96 studies on compliance with hand hygiene guidelines, the global median compliance rate was found to be between 50–60%, suggesting a universal problem [32]. Many healthcare facilities around the world have implemented the WHO’s recommended hand hygiene guidelines, known as Your Five Moments of Hand Hygiene [33]. The five moments of hand hygiene framework emphasizes the need for handwashing at specific points during a clinical session, even when treating the same patient [33]. The Saudi MOH recommends adhering to the WHO guidelines [34]. Our results indicate a lack of knowledge regarding the WHO recommendations, with rates comparable to previous global findings [27,32]. This global lack of knowledge may be influenced by several potential factors. There appears to be limited emphasis on IPC education within healthcare curricula, particularly in developing countries, which might contribute to this issue. Limited access to updated IPC training and resources could further exacerbate the challenge. Additionally, cultural attitudes and potential misconceptions about hand hygiene practices might play a role in hindering compliance and understanding of WHO standards. Institutional challenges, such as staffing shortages, high patient-to-provider ratios, and time constraints, could also contribute to making adherence to IPC guidelines a lower priority in resource-limited healthcare settings. Furthermore, the dissemination of WHO guidelines may not always effectively reach frontline healthcare workers, possibly due to a lack of targeted campaigns or an insufficient focus on practical application in clinical settings. Addressing these challenges could benefit from a multi-faceted approach, including policy adjustments, enhanced training, and improved resource allocation, to help bridge these knowledge gaps globally.

Occupational hazards, particularly sharp injuries, are a major cause of blood-borne diseases like Hepatitis B. Dental practitioners are at a three- to four-fold higher risk of Hepatitis B infection compared to the general population [35]. Sharp injuries are also more likely to occur in dental environments than in other healthcare settings [36]. To reduce the risk of developing transmitted diseases, the MOH vaccination programs have been provided throughout hospitals located in Saudi Arabia [37], and KSAU-HS’s policy mandates that all students and healthcare workers receive needed vaccinations before clinical enrolment to ensure immunity and protection. Hence, this study did not investigate the immunization status of the participants. 

However, this increased risk may be attributed to the nature of dental instruments and the small operating field. In our questionnaire, 189 (87.1%) answered that if they encountered a skin prick injury in the future, they know how to report it. Of participants, 72 (33.18%) experienced a sharp injury while treating a patient, with only 58 (80.55%) of these injuries being reported to the relevant authorities. At King Saud University (KSU), 57% of dental students reported accidental sharp injuries [13], while another study at KSU found that 21.58% of dental students experienced skin injuries [20]. This variation may be attributed to differences in the study population, clinical training environments, or institutional protocols for injury prevention and reporting. Moreover, the disparities in reporting behaviors across studies suggest that awareness and adherence to reporting protocols remain as areas that require further emphasis in dental education and training.

Regarding adherence to IPC protocols, only about half of the participants consistently wash their hands before wearing gloves (54.38%). However, most dental students adhere to wearing PPE, with 82% wearing gowns, 90.78% wearing gloves, and 90.32% wearing masks. These results are comparable to previous studies conducted at KSU [13,20], and another in Jeddah, Saudi Arabia, where PPE compliance ranged between 93.9% to 99.8% [14]. Similar to the findings of Alshiddi et al. (2015) and Halawani et al. (2020), dental students were less concerned with using other protective items, with 74.19% using protective glasses and 36.87% using head caps. The differences in compliance between various PPE items might be attributed to several factors. Items like gloves, gowns, and masks are often emphasized as mandatory in clinical protocols and are more integrated into routine practices. On the other hand, protective glasses and head caps may not be consistently enforced or perceived as critical, leading to reduced adherence. Institutional differences, including the emphasis placed on comprehensive PPE usage during training, accessibility of specific protective equipment, and the perceived risk of exposure, could also explain the variability observed between studies.

Disinfecting prosthodontic tools is crucial to preventing the spread of infections between patients. According to the Occupational Safety and Health Administration guidelines, materials such as mixing spatulas, rubber bowls, or shade guides should be cleaned and disinfected using an intermediate-level hospital tuberculocidal disinfectant [38]. According to one investigation, methicillin-resistant Staphylococcus aureus heavily contaminated the impression guns after routine clinical use, and a combination of disinfection and sterilization were able to reduce bacterial count by 95% [39]. In our study, the percentage of students consistently adhering to IPC practices during prosthodontic procedures ranged from 77.42% to 88.48%. For example, 80.65% to 87.10% of participants reported always cleaning mixing spatulas, rubber bowls, or shade guides after use. Similar results were found in the study by Halawani et al. (2020), where compliance ranged between 79.3% to 82.8% [14]. These values are higher than those reported at KSU (53.5% to 60.5%) and in India (56.1% to 62.2%) [13,40]. However, only 36.87% of participants consistently disinfect the user interface of digital dental scanners between patients, which is a practice not addressed in previous studies. These findings emphasize the importance of expanding IPC training to cover emerging technologies and lesser-emphasized practices to ensure comprehensive infection control in clinical settings.

The risk of cross-infections between dental clinics and laboratories has been documented in numerous studies. A systematic review found that about 60% of impressions and dental items were contaminated when leaving the dental office [41,42]. The Centers for Disease Control (CDC) recommend that all items with potential for contamination be disinfected before being sent to the dental laboratory [43]. Effective communication between dental personnel and laboratory staff is essential to clarify responsibilities for the final disinfection process. According to the CDC, if the dental office does not receive written documentation of the disinfection method used, the dental office is responsible for performing the final disinfection procedures [43]. In our study, around 85% of participants consistently disinfect materials such as impressions, record blocks, and prostheses before sending them to the lab. This is comparable to the findings of Halawani et al. 2020, where 82.6% adhered to disinfection protocols, and is notably higher than the 68% reported by Fahad Alshiddi (2015) [13,14]. Additionally, 82% of participants in our study always disinfect items received from the dental laboratory. This step is particularly important given the findings of Al-Ali et al., who reported poor compliance and a lack of knowledge regarding IPC measures among dental laboratory technicians in Riyadh [44]. 

These findings highlight the need for rigorous disinfection protocols and improved collaboration between dental clinics and laboratories. Enhancing the knowledge and compliance of dental personnel and laboratory technicians through targeted training and documentation processes can reduce the risk of cross-infections.

Self-assessment plays a crucial role in understanding students’ satisfaction with their knowledge and performance regarding IPC during prosthodontic procedures. In our study, most respondents rated their knowledge regarding IPC as excellent (43.32%) or very good (41.94%). This contrasts with the findings of Alshiddi et al. (2015), where only 8.1% rated their knowledge as very good, and the majority evaluated it as good (59.3%) or fair (29.1%) [13]. Similarly, Halawani et al. (2020) reported that almost half of the participants (46.3%) evaluated their knowledge as poor, followed by fair (22%) [14]. This discrepancy could be attributed to the more diverse population in their study and a tendency for self-underestimation among participants [14]. Regarding performance satisfaction, most of our participants were completely satisfied (43.32%) or mostly satisfied (41.01%) with their performance. In comparison, a study conducted at KSU reported that most participants were nearly satisfied (43%) followed by fairly satisfied (36%), while only (8.1%) were completely satisfied [13,14]. Similarly, in Jeddah, the majority of participants were nearly satisfied (40.7%) followed by completely satisfied (24.1%) and fairly satisfied (22.8%) [14].

These variations in self-assessment may stem from differences in curriculum structure, clinical exposure, and training intensity across institutions. Additionally, cultural factors, self-perception, and individual confidence levels could influence how students evaluate their knowledge and performance. 

One limitation of this study is its focus on a single institution, limiting the generalizability of the findings to students and interns from other dental institutions. While the results align with previous local and international studies, variations may be influenced by differences in IPC policies and institutional cultures. Additionally, the questionnaire did not include lab technicians, who play a crucial role in IPC within prosthodontic practice. Sampling-related biases were minimized through stratified random sampling, ensuring representation across academic levels. However, reliance on subjective self-assessments may not accurately capture true knowledge, adherence, or performance, and could introduce recall bias. Furthermore, the online distribution of the survey may not fully reflect actual results. 

Despite these limitations, the findings offer valuable insights for planning and implementing future dental public health policies within this institution and potentially across other dental institutions in the country. By addressing the highlighted gaps and tailoring policies to meet institutional needs, dental education can better align with global IPC standards, ultimately fostering a safer clinical environment and reducing the risk of healthcare-associated infections.

## 5. Conclusions

Our results indicate that dental undergraduate and postgraduate students and interns possess a decent level of knowledge and adherence to IPC practices. Yet, some areas require improvement, which can be addressed through enhanced motivation and education to support IPC measures and boost self-confidence and satisfaction. This study, along with others conducted in the field, can serve as a cornerstone for a national questionnaire among dental schools across the country, aiding in the implementation of standardized organizational policies on IPC guidelines.

## Data Availability

The original contributions presented in this study are included in the article. Further inquiries can be directed to the corresponding author L.A.

## Supplementary Materials

The following supporting information can be downloaded at: https://www.mdpi.com/article/10.3390/healthcare12242536/s1, Knowledge and Compliance with Infection Prevention and Control Practices in Prosthodontic Procedures Among Dental Students and Professionals.

## Appendix A

**Table A1:** Responses on IPC knowledge and practices.

Questions	Correct Answers *n* (%)	Incorrect Answers *n* (%)
Which statement best describes the goal of infection control?	203 (93.55%)	14 (6.45%)
What is the definition of standard precautions?	117 (53.92%)	100 (46.08%)
All patients are potential sources of infection, regardless of their known medical status.	214 (98.62%)	3 (1.38%)
What is the recommended duration for hand washing, in seconds?	148(41.23)	211(58.77)
Hand washing is necessary between clinical tasks and procedures on the same patient.	128 (58.99%)	89 (41.01%)
Gloves should be changed between different clinical procedures on the same patient.	147 (67.74%)	70 (32.26%)
Masks should be replaced between different clinical procedures on the same patient.	68 (31.34%)	149 (68.66%)
Isolation methods such as rubber dam and cotton roll, are crucial for infection control.	184 (84.79%)	33 (15.21%)
Gloves should be changed after treating each patient.	215 (99.08%)	2(0.92%)
Masks should be changed after treating each patient.	209 (96.31%)	8 (3.69%)
If you were to encounter a skin prick injury in the future, you would know how and where to report it.	189 (87.10%)	28(12.90%)

**Table A2:** Adherence of participants with IPC in dental practice.

Item	Always	Sometimes	Never
Do you wash your hands before wearing gloves?	118 (54.38%)	99 (45.62%)	0
Do you wash your hands after treating each patient?	176 (81.11%)	41 (18.89%)	0
Do you wear gloves during patient treatment?	197 (90.78%)	20 (9.22%)	0
Do you change your gloves after treating each patient?	202 (93.09%)	15 (6.91%)	0
Do you wear a face mask during patient treatment?	196 (90.32%)	21 (9.68%)	0
Do you replace your face mask after treating each patient?	155 (71.43%)	62 (28.57%)	0
Do you wear a protective face shield or eye goggle during patient treatment?	118 (54.38%)	99 (45.62%)	0
Do you disinfect the face shield or eye goggles after treating each patient?	161 (74.19%)	56 (25.81%)	0
Do you provide the patient with protective eyewear during treatment?	88 (40.55%)	129 (59.45%)	0
Do you wear a protective gown during patient treatment?	178 (82.03%)	39 (17.97%)	0
Do you change your protective gown after treating each patient?	181 (83.41%)	36 (16.59%)	0
Do you wear a head cap during patient treatment?	80 (36.87%)	137 (63.13%)	0
Do you change your head cap after treating each patient?	123 (56.68%)	94 (43.32%)	0
Do you use protective barriers (on the light handle, clinic’s screen, and keyboard) in the clinic?	201 (92.63%)	16 (7.37%)	0
Do you (or your assistant) change the barriers after treating each patient?	208 (95.85%)	9 (4.15%)	0
Do you use a rubber dam for isolation during procedures?	165 (76.04%)	52 (23.96%)	0
Do you remove your watch or jewelry before treating patients?	123 (56.68%)	94 (43.32%)	0
Do you (or your assistant) disinfect the dental clinic after treating each patient?	205 (94.47%)	12 (5.53%)	0

**Table A3:** Adherence of participants with IPC in prosthodontic clinical practice.

Item	Always	Sometimes	Never
Do you (or your assistant) disinfect the rubber bowl between patients?	189 (87.10%)	28 (12.90%)	0
Do you (or your assistant) disinfect the mixing spatula between patients?	181 (83.41%)	36 (16.59%)	0
Do you (or your assistant) sterilize/disinfect the face bow between patients?	180 (82.95%)	37 (17.05%)	0
Do you (or your assistant) disinfect the shade guide between patients?	175 (80.65%)	42 (19.35%)	0
Do you (or your assistant) sterilize/disinfect the wax knife/torch between patients?	170 (78.34%)	47 (21.66%)	0
Do you (or your assistant) disinfect the impression gun between patients?	172 (79.26%)	45 (20.74%)	0
Do you (or your assistant) rinse the impression before sending it to the lab?	192 (88.48%)	25 (11.52%)	0
Do you (or your assistant) disinfect the laboratory work (impression, record block, prosthesis etc.) before sending it to the lab?	185 (85.25%)	32 (14.75%)	0
Do you (or your assistant) sterilize/disinfect impression trays (stock, custom and metal) before using them?	168 (77.42%)	48 (22.12%)	1 (0.46%)
Do you (or your assistant) disinfect the laboratory work (impression, record block, prosthesis etc.) before placing it into the patient’s mouth?	178 (82.03%)	39 (82.03%)	0
Do you (or your assistant) change the intra oral scanner tip between patients?	187 (86.18%)	30 (13.82%)	0
Do you (or your assistant) disinfect the scanning wand, mouse, keyboard, and touch screen between patients?	80 (82.03%)	137 (63.13%)	0
