# Supplementary material for: Knowledge and Compliance with Infection Prevention and Control Practices in Prosthodontic Procedures Among Dental Students and Professionals
Source: Healthcare (Basel). 2024 Dec 16;12(24):2536. doi: 10.3390/healthcare12242536 (PMC11675545; doi:10.3390/healthcare12242536)
Supplement: Supplementary file 1 [file healthcare-12-02536-s001.zip › healthcare-3343347-supplementary.pdf]

NRC23R/557/0

King Abdullah International Medical Research Center
KAIMRC
APPROVED
IRB
Institutional Review Board

Knowledge and Compliance with Infection Prevention and Control Practices in Prosthodontic Procedures Among Dental Students and Professionals

Section A: Demographic Data of Participants

1. Sex:
   a. Male
   b. Female

2. Academic level:
   a. D3 Student
   b. D4 Student
   c. Intern
   d. Postgraduate Resident

Section B: Knowledge of Participants Regarding Infection Prevention and Control

1. Which statement best describes the goal of infection control [1]?
   a. Preventing or stopping the spread of infection
   b. Protecting patients from infection
   c. Protecting healthcare personnel from infection
   d. I don't know
2. What is the definition of standard precautions [1]?
   a. Minimum infection prevention practices applied to all patient care
   b. A set of personal protective equipment worn by healthcare providers
   c. Basic level of measures used when treating patients with confirmed/suspected infectious disease
   d. I don't know
3. All patients are potential sources of infection, regardless of their known medical status [1].
   a. True
   b. False
4. What is the recommended duration for hand washing, in seconds? *
   a. 10-30
   b. 40-60
   c. 60-80
   d. More than 80
5. Hand washing is necessary between clinical tasks and procedures on the same patient [1].
   a. True
   b. False
6. Gloves should be changed between different clinical procedures on the same patient [1].

a. True

b. False

7. Masks should be replaced between different clinical procedures on the same patient [1].
   a. True
   b. False

8. Isolation methods such as rubber dam and cotton roll, are crucial for infection control [2].

   a. True

   b. False

King Abdullah International Medical Research Center
KAIMRC
APPROVED
IRB
Institutional Review Board

9. Gloves should be changed after treating each patient. *
   a. True
   b.
   c. False
10. Masks should be changed after treating each patient. *
   a. True
   b. False
11. If you were to encounter a skin prick injury in the future, you would know how and where to report it. *
   a. True
   b. False

King Abdullah International Medical Research Center
KAIMRC
APPROVED
IRB
Institutional Review Board

Section C: **Adherence of Participants with IPC in Dental Practice.**

Item	Always	Sometimes	Never
Do you wash your hands before wearing gloves [3,4]?			
Do you wash your hands after treating each patient [3,4]?			
Do you wear gloves during patient treatment [3,4]?			
Do you change your gloves after treating each patient [3,4]?			
Do you wear a face mask during patient treatment [3,4]?			
Do you replace your face mask after treating each patient [3,4]?			
Do you wear a protective face shield or eye goggle during patient treatment [3,4]?			
Do you disinfect the face shield or eye goggles after treating each patient [3,4]?			
Do you provide the patient with protective eyewear during treatment [3,4]?			
Do you wear a protective gown during patient treatment [3,4]?			
Do you change your protective gown after treating each patient [3,4]?			
Do you wear a head cap during patient treatment [3,4]?			
Do you change your head cap after treating each patient [3,4]?			
Do you use protective barriers (on the light handle, clinic's screen, and keyboard) in the clinic [3,4]?			
Do you (or your assistant) change the barriers after treating each patient [3,4]?			
Do you use a rubber dam for isolation during procedures [3,4]?			
Do you remove your watch or jewelry before treating patients [3,4]?			
Do you (or your assistant) disinfect the dental clinic after treating each patient [3,4]?			

1. Have you ever encountered a skin prick injury from a sharp instrument while treating a patient [2]?
    a. Yes
    b. No
2. If you answered 'Yes' to the previous question, did you report the injury [1]?
    a. Yes
    b. No

King Abdullah International Medical Research Center
KAIMRC
APPROVED
IRB
Institutional Review Board

Section D: Adherence of Participants with IPC in Prosthodontic Clinical Practice.

Item	Always	Sometimes	Never
Do you (or your assistant) disinfect the **rubber bowl** between patients [5]?			
Do you (or your assistant) disinfect the **mixing spatula** between patients [5]?			
Do you (or your assistant) sterilize/disinfect the **face bow** between patients [5]?			
Do you (or your assistant) disinfect the **shade guide** between patients [5]?			
Do you (or your assistant) sterilize/disinfect the **wax knife/torch** between patients [6]?			
Do you (or your assistant) disinfect the **impression gun** between patients [6]?			
Do you (or your assistant) rinse the **impression** before sending it to the lab [6]?			
Do you (or your assistant) disinfect the **laboratory work** (impression, record block, prosthesis etc.) before sending it to the lab [6]?			
Do you (or your assistant) sterilize/disinfect **impression trays** (stock, custom and metal) before using them [7]?			
Do you (or your assistant) disinfect the **laboratory work** (impression, record block, prosthesis etc.) before placing it into the patient's mouth [6]?			
Do you (or your assistant) change the **intra oral scanner tip** between patients? *			
Do you (or your assistant) disinfect the **scanning wand, mouse, keyboard, and touch screen** between patients?*			

Section E: Self Evaluation

1. How would you evaluate your knowledge regarding infection prevention and control in the prosthodontic clinic [5]?
    a. Excellent
    b. Very good
    c. Adequate
    d. Limited
    e. Insufficient
2. How satisfied are you with your knowledge and performance in the area of infection prevention and control within prosthodontic clinical settings [5]?
    a. Completely satisfied
    b. Mostly satisfied
    c. Somewhat satisfied

d. Slightly satisfied
e. Not satisfied

References:

1- Khubrani A, et al. Knowledge and information sources on standard precautions and infection control of Health Sciences students at King Saud bin Abdulaziz University for Health Sciences, Saudi Arabia, Riyadh. *J Infect Public Health* **2018,** 11, *546–549.*

2- Alharbi, G.; Shono, N.; Alballaa, L.; Aloufi, A. Knowledge, attitude, and compliance of infection control guidelines among dental faculty members and students in KSU. *BMC Oral Health* **2019**, *19, 1-8*.

3- Al-Essa NA, AlMutairi MA. To what extent do dental students comply with infection control practices? *Saudi J Dent Res* **2017,** 8, *67–72.*

4- 23. Al-Maweri SA, Tarakji B, Shugaa-Addin B, Al-Shamiri HM, Alaizari NA, AlMasri O. Infection control: Knowledge and compliance among Saudi undergraduate dental students. *GMS Hyg Infect Control* **2015,** *10.*

5- Alshiddi, I.F. Attitude and Awareness of Dental Students and Interns toward Infection Control Measures in Prosthodontic Clinics. *J Int Oral Health* **2015**, *7*, *10–5.*

6- Rehman, B.; Bano, V.; Afzal, S.; Arshad, M.; Rehan, A.; Mustafa, S. Awareness and Practice of Cross Infection Control in Prosthodontics Department among Undergraduates, Graduates, and Postgraduates in a Tertiary Dental Health Care Institution. *Life Sci* **2022**, *3, 7–7.*

7- Halawani, R.; Aboalshamat, K.; Alwsaidi, R.; Sharqawi, S.; Alhazmi, R.; Abualsaud, Z., et al. Awareness and Practices of Dental Students and Dentists Regarding Infection Control in Prosthodontic Clinics. *Open Dent J* **2020**, *14*, *184–90.*

* Original questions that are not taken from other questionnaires.
